# Demonstration of a refractometric sensor based on an optical micro-fiber three-beam interferometer

**DOI:** 10.1038/srep07504

**Published:** 2014-12-16

**Authors:** Chunyang Han, Hui Ding, Fangxing Lv

**Affiliations:** 1 State Key laboratory of Electrical Insulation and Power Equipment, Xi'an Jiaotong University, Xi'an 710049, China

## Abstract

With diameter close to the wavelength of the guided light and high index contrast between the fiber and the surrounding, an optical micro-fiber shows a variety of interesting waveguiding properties, including widely tailorable optical confinement, strong evanescent fields and waveguide dispersion. Among various micro-fiber applications, optical sensing has been attracting increasing research interest due to its possibilities of realizing miniaturized fiber optic sensors with small footprint, high sensitivity, and low optical power consumption. Typical micro-fiber based sensing structures, including Michelson interferometer, Mach-Zenhder interferometer, Fabry-Perot interferometer, micro-fiber ring resonator, have been proposed. The sensitivity of these structures heavily related to the fraction of evanescent field outside micro-fiber. In this paper, we report the first theoretical and experimental study of a new type of refractometric sensor based on micro-fiber three-beam interferometer. Theoretical and experimental analysis reveals that the sensitivity is not only determined by the fraction of evanescent field outside the micro-fiber but also related to the values of interferometric arms. The sensitivity can be enhanced significantly when the effective lengths of the interferometric arms tends to be equal. We argue that this has great potential for increasing the sensitivity of refractive index detection.

Micro-fiber based refractometric sensors are currently in the spotlight of research as basic functional elements for physical^1^,^2^,^3^, chemical^4^,^5^ and biological^6^,^7^ sensing. Due to the small dimension for light confinement, a wave-guiding micro-fiber leaves a considerable fraction of the guided field outside the micro-fiber as evanescent waves, making it sensitivity to the change of ambient refractive index (RI). During the past few years, a number of micro-fiber based refractometric sensors have be developed using Michelson interferometer^8^, Mach-Zenhder interferometer^9^,^10^, Fabry-Perot interferometer^11^, micro-fiber ring resonator^12^,^13^, and micro-fiber coil resonator^14^, *et al.* The sensitivity of these types of sensor relies on the interaction between the evanescent field and and external medium. It is believed that the most feasible approach to improve the sensitivity is to enhance the evanescent wave^15^,^16^. Many efforts have previously been made by decreasing the radius of micro-fiber^17^, twisting or bending the micro-fiber^18^, *et al.* However, precise adjustment of evanescent field is a challenge task in practice.

In this paper, we propose a new type of micro-fiber based refractometric sensor by using three-beam interferometer (TBI) construction. Comparing with the conventional micro-fiber based refractometric sensors, the sensitivity of the proposed sensor is not only originated from the evanescent field outside the fiber but also determined by the lengths of the two arms of the proposed interferometer. The sensitivity can be enhanced significantly when the effective length difference of the two arms approaches zero. This suggests a new technical means for increasing the sensitivity. The operating principle and theoretical model of the TBI will be explained in the context of its use as a refractometric sensor. The variation of sensitivity with the fraction of evanescent field and the lengths of interferometric arms will be highlighted. In addition, we will demonstrate our discovery by experimental results.

## Results

### Concept and operating principle of TBI

**Figure 1:**
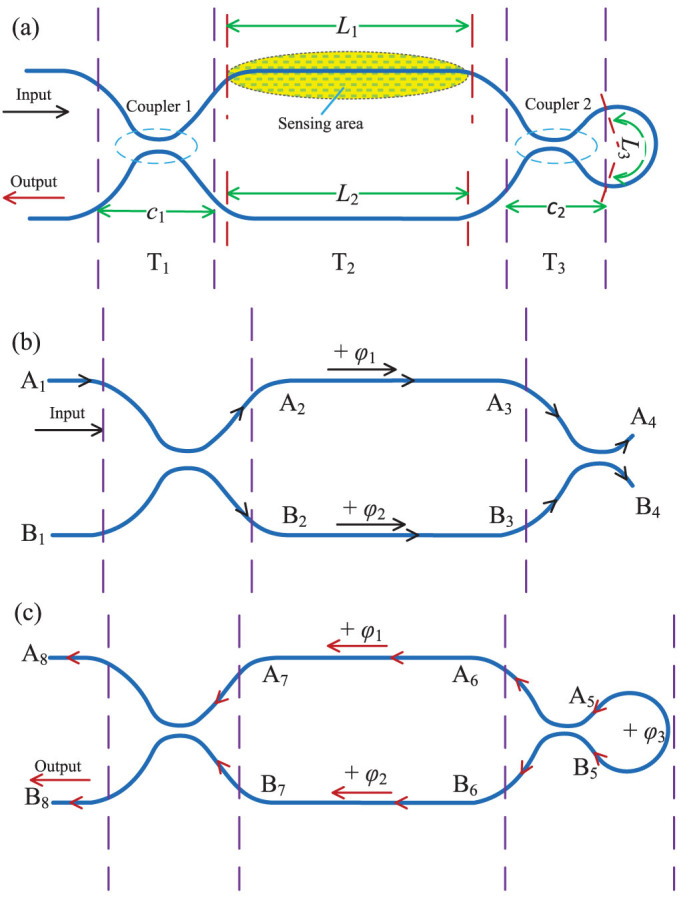
(a) Schematic design for micro-fiber based three-beam interferometer architecture. *T*_1_, *T*_2_, snd *T*_3_ stand for the transformation matrix in different sections of TBI. *c*_1_ and *c*_2_ represent the effective lengths of the first and the second coupler, respectively. (b) Schematic illustration of the light wave forward propagation in the TBI. The black arrow indicates the direction of light propagation. (c) Schematic illustration of the light wave backward propagation in the TBI. The red arrow indicates the direction of light propagation.

**Figure 2:**
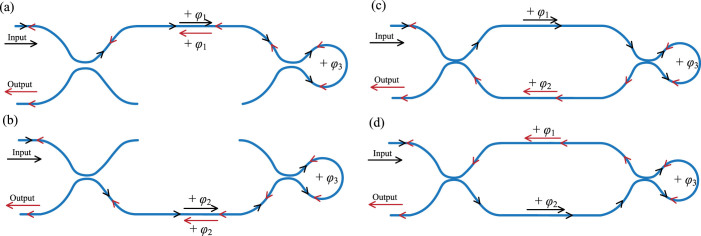
Schematic of the four paths in the proposed TBI. The arrow indicates the direction of light propagation.

Figure 1(a) shows schematically the proposed TBI. The interferometer consists of a Mach-Zehnder interferometer and a Sagnac loop. The operating principle can be explained with the aid of Fig. 1 and Fig. 2. When the incident light is launched into the input port of TBI as shown in Fig. 1(b), this beam will be split into two by the first coupler. The two resultant beams are transmitted and reected by the second coupler and Sagnac loop, and then back to the first coupler. The total light field at the output port of TBI is a summation of four components corresponding the four paths as shown in Fig. 2. Since the third path (as shown in Fig. 2(c)) and the fourth path (as shown in Fig. 2(d)) are the same, light beams travelling in these two paths experience the same phase delay and phase shift, thus the total light field at the output port of the TBI is a summation of three components, exactly.

### Theoretical model of TBI

The optical paths followed by the incident light are indicated by the arrows shown in Fig. 1(b) and Fig. 1(c). We consider the case in which an optical beam with power 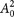 is injected into the input port of the TBI (as shown in Fig. 1(b)). Here we assume that both of the upper and lower arms have the same waveguide structures; that is, the outputs of the first coupler are given by^19^,^20^

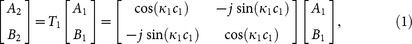
where *T*_1_ is the transfer matrix of the first coupler, *A*_1_ = *A*_0_, *B*_1_ = 0. *κ*_1_ and *c*_1_ denote the coupling coefficient and coupling length of the first coupler, respectively.

After passing through the straight arms of TBI, *A*_3_ and *B*_3_ become 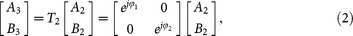
where *T*_2_ is a diagonal matrix which represents the phase shift in the straight region of TBI (as shown in Fig. 1(a)). *ϕ*_1_ and *ϕ*_2_ stand for the phase shift in the upper and lower arms (as shown in Fig. 1(b) and Fig. 1(c)), respectively. The phase shift *ϕ*_1_ and *ϕ*_2_ can be expressed as 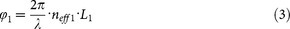
and 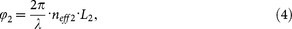
where *n_eff_*_1_ and *n_eff_*_2_ are the effective index of the propagating mode guided along the upper and lower arms. *L*_1_ and *L*_2_ are the lengths of the two arms (as shown in Fig. 1(a)). *λ* denotes the wavelength of light in vacuum.

Then the output of the second coupler *A*_4_ and *B*_4_ can be obtained by 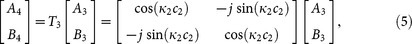
where *κ*_2_ and *c*_2_ denote the coupling coefficient and coupling length of the second coupler, respectively. *T*_3_ is the transfer matrix of the second coupler.

When lights enter into the loop, the lights will pass through the loop and back to the right ends of the second coupler (as shown in Fig. 1(c)). During the lights passing through the loop, the two beams of light in opposite angular directions experience a same phase delay 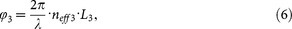
where *n_eff_*_3_ denotes the effective index of the propagating mode guided along the loop and *L*_3_ represents the length of the loop.

Then *A*_5_ and *B*_5_ (as shown in Fig. 1(c)) can be expressed as^21^

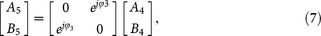
When the reected lights launched from the right ends of the second coupler, the lights will propagate along the micro-fiber and finally arrive at the output port of the proposed TBI. The overall transformation matrix for this progress can be computed by concatenating the three transfer matrices *T*_1_, *T*_2_, and *T*_3_. Therefore, the light field *A*_8_ and *B*_8_ can be calculated by 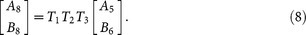
Combining Eqs. (1) to (8), we obtain the light field at the output port, *B*_8_, as 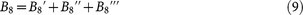
where 
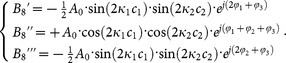


It can be seen from the above equations that the output light field is a summation of three field components, which is in accordance with the operating principle analyzed in the former section.

Here, we may note the following special cases:

- *One of κ*_1_*c*_1_ *and κ*_2_*c*_2_ *is *
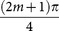
* and the other one is not* 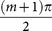
*, m is a positive integer, simultaneously.* In this case, from Eq. (9) we can find that the component 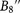 is zero. Thus the output field *B*_8_ only contain two components (as shown in Fig. 2(a) and Fig. 2(b)), the proposed TBI becomes a Michelson interferometer.
- *One of κ*_1_*c*_1_ *and κ*_2_*c*_2_ *equals to *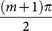. In this case, 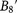 and 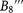 become zero. This result indicates that the TBI behaves like an optical switch. When the other of *κ*_1_*c*_1_ and *κ*_2_*c*_2_ equals to 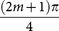 the resulting output field *B*_8_ is zero. Otherwise 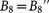, represents the output light contain only one component.

However, we are not concerned with the above particular cases. The work of this paper, as an effort and achievement on the route of improvement the sensing sensitivity of micro-based refractometric sensors, focuses on the issues of the operating principle and theoretical model of the proposed TBI as a three-beam interferometer.

**Figure 3:**
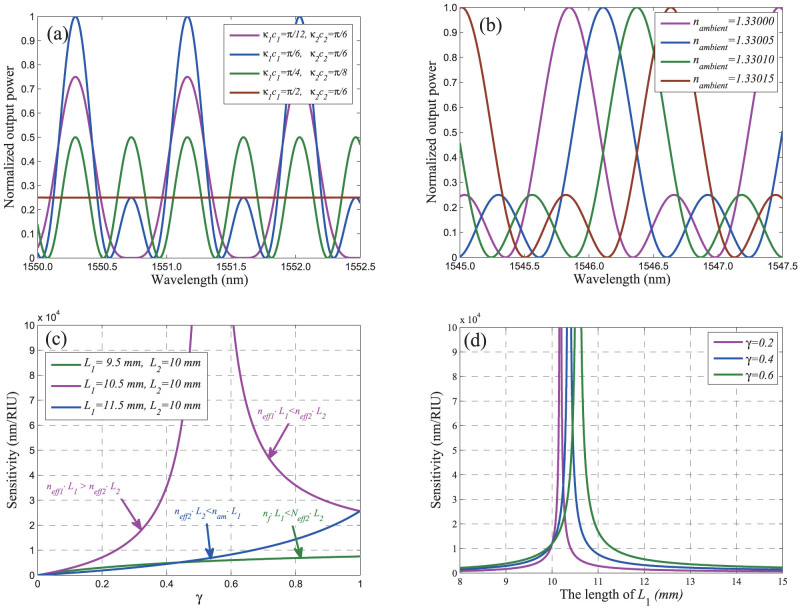
(a) The calculated output spectra from output port of TBI under different *κ*_1_ · *c*_1_ and *κ*_2_ · *c*_2_. (b) The calculated output spectra of TBI with different ambient RIs. The parameters of TBI are *γ* = 0.5, *κ*_1_ · *c*_1_ = *κ*_2_ · *c*_2_ = *π*/6, *L*_1_ = 10 *mm*, and *L*_2_ = 9 *mm*. (c) Theoretical sensitivity of TBI as a function of the fraction of evanescent field outside the micro-fiber under different values of *L*_1_ and *L*_2_. The ambient RI is around 1.33000 and the RIU here is the abbreviation for refractive index unit. (d) Dependence of the sensitivity of TBI for RI sensing on *L*_1_ under the condition *L*_2_ = 10 *mm*.

The total light intensity at the output port of TBI can be obtained from Eq. (9)

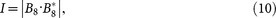
where 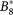 is the complex conjugate of *B*_8_. The output spectrum as a function of wavelength is depicted in Fig. 3(a).

### Refractive index sensitivity of TBI

If the RI of the environment surrounding the micro-fiber in the sensing area (as shown in Fig. 1(a)) is changed, the effective index *n_eff_*_1_ will be changed accordingly, which will result in the shifts of the output spectra. Figure 3(b) plots several output spectra under different ambient RIs. From the figure we can find the peak-wavelength will shift with the variation of ambient RI. For the ambient RI measurement using spectral shift, the sensitivity can be defined as 
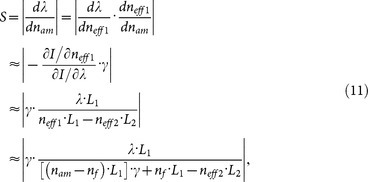
where *n_am_* and *n_f_* denote the RI of ambient medium and micro-fiber, respectively, and *γ* is a value in the range among 0 and 1 which characterizes the fraction of the evanescent field outside the micro-fiber. For the slight variations of the RI of ambient materials, *γ* can be approximately regarded as a constant.

As shown in Eq. (11), the sensitivity is not only determined by the fraction of evanescent field, *γ*, but also related to the values of *L*_1_ and *L*_2_. The sensitivity as a function of the fraction of evanescent, *γ*, is illustrated in Fig. 3(c). From this figure, we can see:

- If and only if *n_f_* · *L*_1_ < *n_eff_*_2_ · *L*_2_ or *n_eff_*_2_ · *L*_2_ < *n_am_* · *L*_1_ the sensitivity will increase with the fraction of evanescent field, *γ*, monotonously;
- In the case of *n_am_* · *L*_1_ < *n_eff_*_2_ · *L*_2_ < *n_f_* · *L*_1_, the sensitivity increases with *γ* when *n_eff_*_1_ · *L*_1_ > *n_eff_*_2_ · *L*_2_ and decreases under the condition *n_eff_*_1_ · *L*_1_ < *n_eff_*_2_ · *L*_2_;
- The sensitivity can be enhanced significantly when the effective lengths of the two arms of TBI approaches equal (here, we define *n_eff_*_1_ · *L*_1_ and *n_eff_*_2_ · *L*_2_ as the effective lengths of the upper and lower arms of TBI, respectively).

The above theoretical analysis shows that the sensitivity of the proposed TBI is not only determined by the fraction of evanescent field, but also related to the values of *L*_1_ and *L*_2_. Fig. 3(d) indicates the sensitivity of TBI as the function of *L*_1_ under the condition *γ* and *L*_2_ are constant. We can find that the sensitivity increases rapidly as the length of *L*_1_ tends to 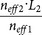.

In general, precise adjustment of the fraction of evanescent field is challenging task. As a result the sensitivity of micro-fiber based RI sensor can hardly meet the requirement by adjusting *γ*. However, precise adjustment of *L*_1_ and *L*_2_ can be achieved quite easily. This suggests a new technical means for increasing the sensitivity of micro-fiber based refractometric sensor.

**Figure 4:**
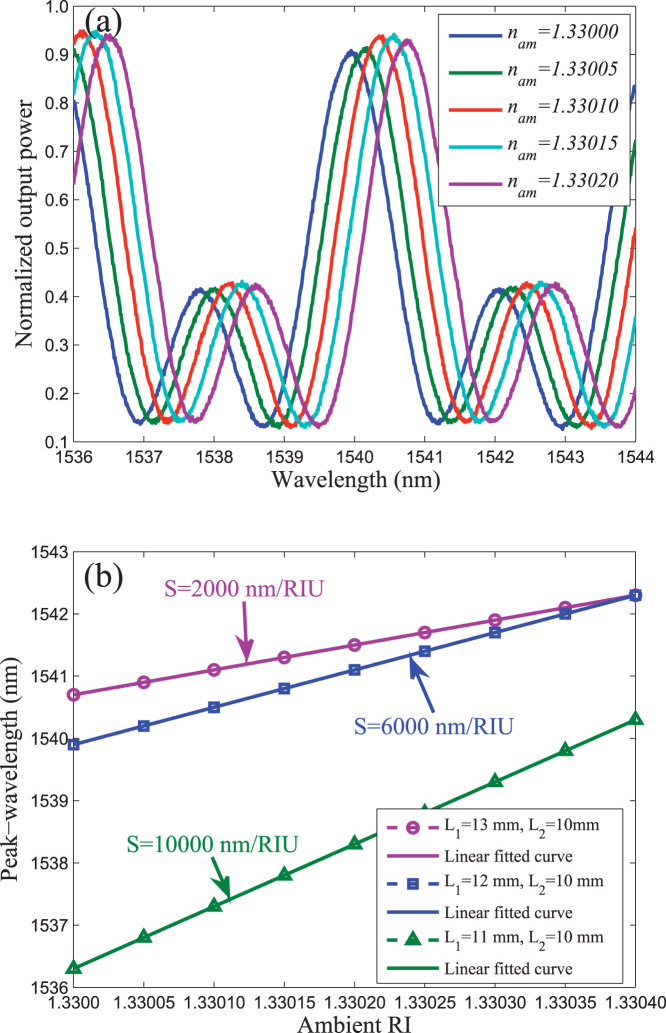
(a) Output spectra of TBI in aqueous solution of alcohol with five different concentrations. The values of *L*_1_ and *L*_2_ are 12 *mm* and 10 *mm*, respectively. (b) Response of peak-wavelength shift to ambient RI change from 1.33000 to 1.33020 for different values of *L*_1_ and *L*_2_.

We further demonstrate our discovery by experiments. We measure the spectral response of the TBI to ambient RI by immersing the upper arm into an aqueous solution of alcohol (as shown in Fig. 1(a)), with the solution's RI modified by changing the concentration of alcohol. Figure 4(a) gives the measured peak-wavelengths as a function of ambient RI ranging from 1.33000 to 1.33020. Figure 4(b) records the relationship between the peak-wavelength and ambient RI under difference values of *L*_1_ under the condition *L*_2_ remains a constant value. Comparing the sensitivity of the proposed TBI in different values of *L*_1_, we can see that when *L*_1_ is 3 *mm* longer than *L*_2_, the sensitivity is only 2000 nm/RIU (refractive index unit). With the decrease of the value of *L*_1_, the sensitivity will become higher and higher. For instance, when *L*_1_ decreased to be 11 *mm*, the sensitivity achieve to 10 000 nm/RIU. This reveals that the sensitivity of TBI heavily relies on the values of *L*_1_ and *L*_2_. The experimental results are in accordance with the theoretical analysis.

## Discussion

For the measurement of an ambient RI change of Δ*n_am_*, the corresponding peak-wavelength shift Δ*λ* is given by 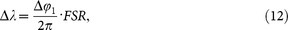
where *FSR* stands for the free spectral range of the output spectrum of proposed TBI.

From Eq. (12) we can see that the sensitivity of TBI to ambient RI changes depends on two factors: the phase changes Δ*ϕ*_1_, and the free spectral range of the output spectrum *FSR*.

The phase changes Δ*ϕ*_1_ associated with variations in ambient refractive index, Δ*n_am_*, is given by 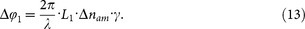
From Eq. (12) and Eq. (13), we may get the conclusion that the sensitivity can be increased by increasing the fraction of evanescent *γ*. However, as shown in Fig. 3(c), the sensitivity varies non-monotonically with *γ* when *n_am_* · *L*_1_ < *n_eff_*_2_ · *L*_2_ < *n_f_* · *L*_1_. Therefore, in general, higher sensitivity cannot be obtained simply by increasing *γ*. This is mainly due to the fact that *FSR* of the TBI also related to *γ*. From Eq. (9) and Eq. (10), the *FSR* can be expressed as 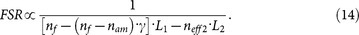
As shown in Eq. (14), *FSR* may decreases as *γ* increased. Consequently, from Eq. (12) we can see that it is difficult to get the conclusion whether the sensitivity is increased according to the increase of *γ*. This is the reason why the sensitivity increases with *γ* when *n_eff_*_1_ · *L*_1_ > *n_eff_*_2_ · *L*_2_ and decreases under the condition *n_eff_*_1_ · *L*_1_ < *n_eff_*_2_ · *L*_2_ in the case of *n_am_* · *L*_1_ < *n_eff_*_2_ · *L*_2_ < *n_f_* · *L*_1_ (as shown in Fig. 3(c)). If *γ* satisfies a certain condition to make *n_eff_*_1_ · *L*_1_ ≈ *n_eff_*_2_ · *L*_2_, the *FSR* will reach its maximum value. Consequently, the sensitivity can be enhanced significantly. However, it would be a challenging to fabricate such a micro-fiber with desired *γ* in practice.

**Figure 5:**
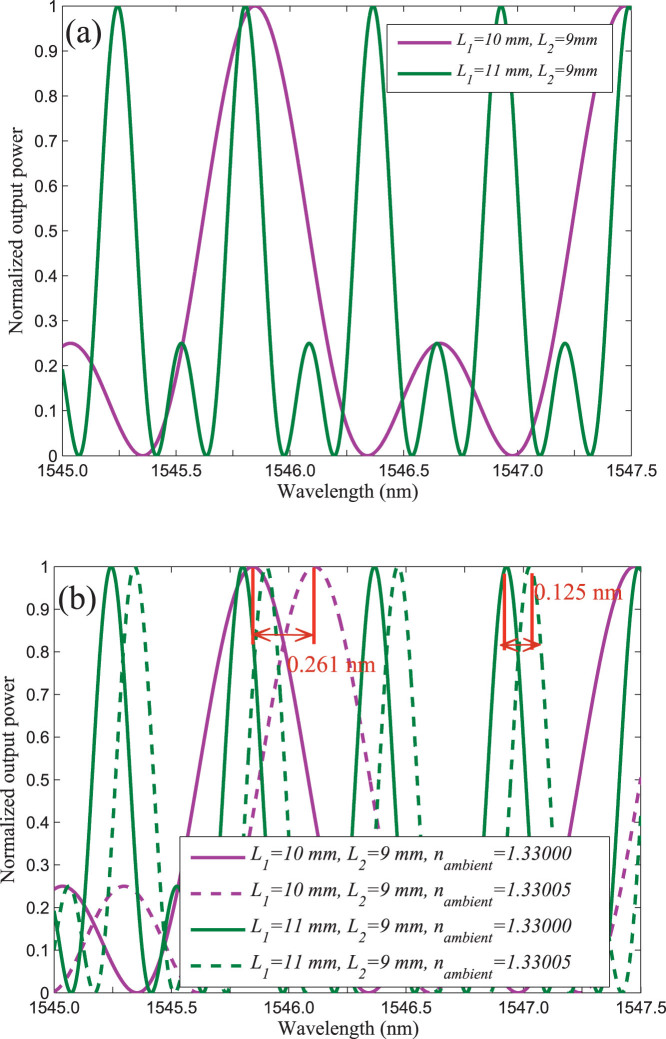
(a) The output spectra of TBI under different values of *L*_1_ and *L*_2_ when the fraction of evanescent *γ* equals to 0.5 and keep constant; (b) The output spectra of TBI under different ambient RIs.

If the the fraction of evanescent field *γ* remain unchanged, the phase changes Δ*ϕ*_1_ associated with Δ*n_am_* will be a constant. Therefore the sensitivity of TBI only determined by *FSR*. In this case, the sensitivity can be easily enhanced by regulating the lengths of refractometric arms of TBI. When the effective lengths of the two arms approaches equal, the *FSR* will become significantly large. Consequently, the sensitivity can be enhanced significantly. Figure 5(a) depicts the spectra of two TBI under different values of *L*_1_ and *L*_2_ under the condition the fraction of evanescent equals to 0.5. From this figure we can find that the *FSR* is heavily rely on the values of *L*_1_ and *L*_2_. Figure 5(b) shows the output spectra of the two TBI under different ambient RIs. Comparing the peak-wavelength changes of the two TBI, we can see that under the condition of *L*_1_ = 11 *mm* and *L*_2_ = 9 *mm*, the peak-wavelength changes 0.125 *nm* when the ambient RI changes from 1.33000 to 1.33005. When *L*_1_ = 10 *mm* and *L*_2_ = 9 *mm*, the peak-wavelength changes 0.261 *nm* as the ambient RI changes from 1.33000 to 1.33005. This means that the sensitivity will be doubled when the values of *L*_1_ decreases from 11 *mm* to 10 *mm*. If we further decrease *L*_1_, the sensitivity will be further improved. When the values of *L*_1_ and *L*_2_ satisfied the condition *n_eff_*_1_ · *L*_1_ ≈ *n_eff_*_2_ · *L*_2_, the sensitivity of TBI will be enhanced significantly. Figure 3(d) indicates the dependence of the sensitivity of TBI for RI sensing on the values of *L*_1_ and *L*_2_. We can see from the figure that the sensitivity increases rapidly as the length of *L*_1_ tends to 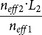. From the above analysis, we can find that the sensitivity can be enhanced significantly by adjusting the lengths of the two arms of TBI.

In conclusion, a refractometric sensor based on a micro-fiber three-beam interferometer was theoretically and experimentally demonstrated. In contrast to the earlier proposed micro-fiber based refractometric sensor, we have discovered that the sensitivity of this type of sensor is not only determined by the fraction of evanescent field outside micro-fiber but also related to the lengths of TBI's arms. The sensitivity can be enhanced significantly when the effective length difference of the two arms approaches zero. This suggests a new means for increasing the sensitivity. It can be foreseen that a micro-fiber based refractometric sensor using TBI architecture may be beneficial for improving the sensitivity significantly by adjusting the lengths of both arms of TBI.

## Methods

### Fabrication and package of TBI

**Figure 6:**
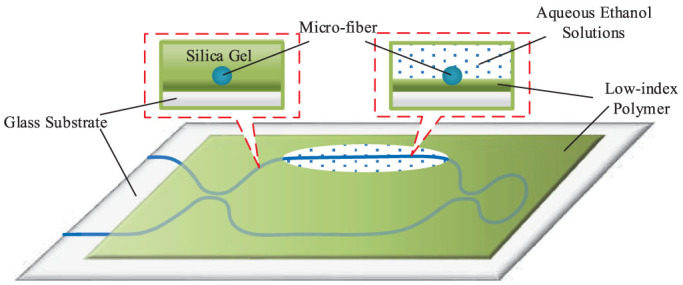
Schematic diagram of the Packaged micro-fiber three-beam interferometer.

First a micro-fiber with 1 *μm* in radius and 65 *mm* in length was fabricated from a standard single mode fiber (SMF-28, Corning) using the ame brushing method^16^,^22^. Then, the architecture of the proposed TBI as shown in Fig. 1(a) was constructed by adopting the micro-fiber. A plate of glass is adopted as the substrate to support the proposed TBI and ambient medium. In order to avoid evanescent field leaking from TBI into the glass substrate whose RI is higher than micro-fiber, a layer of low-index polymer (here, we use silica gel as the polymer, the RI of silica gel is 1.401, which is smaller than micro-fiber and glass substrate) was first coated on the glass plate. After this low-index polymer is cured, we put the fabricated TBI on the substrate. Then we coated the TBI expect for the sensing region by the same silica gel. After the silica is cured, the package is complete. The package would not only make the TBI robust, but also ensure the evanescent field is able to access the external medium. Figure 6 shows the schematic diagram of the packaged TBI.

### Description of experimental setup

The spectral characterization of TBI was carried out by connecting a broadband light source (SLED 1550) to the input port and an optical spectrum analyzer (Yokogawa AQ6370B) to the output port. We measure the spectral response of the TBI to ambient RI by immersing the upper arm into an aqueous solution of alcohol (as shown in Fig. 6), with the solution's RI modified by changing the concentration of alcohol. During the experiment, the temperature of laboratory is kept constant (25°C).

## Author Contributions

D.H. supervised the project; H.C.Y. developed the concept and conceived the design; H.C.Y. performed the simulation and calculation; D.H., H.C.Y. and L.F.X. performed the experiments; L.F.X. prepared the samples; H.C.Y. and D.H. discussed the results and wrote the manuscript; All authors reviewed the manuscript.

## Supplementary Material

Supplementary Information
